# Improved intra-array and interarray normalization of peptide microarray phosphorylation for phosphorylome and kinome profiling by rational selection of relevant spots

**DOI:** 10.1038/srep26695

**Published:** 2016-05-26

**Authors:** Jetse Scholma, Gwenny M. Fuhler, Jos Joore, Marc Hulsman, Stefano Schivo, Alan F. List, Marcel J. T. Reinders, Maikel P. Peppelenbosch, Janine N. Post

**Affiliations:** 1 Department of Developmental BioEngineering, MIRA institute for biomedical technology and technical medicine, University of Twente, P.O. Box 217, NL-7500 AE Enschede, The Netherlands; 2 Department of Gastroenterology and Hepatology. Erasmus MC, University Medical Center Rotterdam, ’s Gravendijkwal 230, NL-3015 CE Rotterdam, The Netherlands; 3 Pepscope BV, Dantelaan 83, 3533 VB Utrecht, The Netherlands; 4 Department of Clinical Genetics, VU University Medical Center, 1007 MB Amsterdam, The Netherlands; 5 Delft Bioinformatics Lab. Delft University of Technology, Mekelweg 4, NL-2628 CD Delft, The Netherlands; 6 Department of Formal Methods and Tools, CTIT institute, University of Twente, P.O. Box 217, NL-7500 AE Enschede, The Netherlands; 7 Department of Malignant Hematology, Lee Moffitt Cancer Center and Research Institute, 12902 Magnolia Drive, Tampa, FL 33612, USA

## Abstract

Massive parallel analysis using array technology has become the mainstay for analysis of genomes and transcriptomes. Analogously, the predominance of phosphorylation as a regulator of cellular metabolism has fostered the development of peptide arrays of kinase consensus substrates that allow the charting of cellular phosphorylation events (often called kinome profiling). However, whereas the bioinformatical framework for expression array analysis is well-developed, no advanced analysis tools are yet available for kinome profiling. Especially intra-array and interarray normalization of peptide array phosphorylation remain problematic, due to the absence of “housekeeping” kinases and the obvious fallacy of the assumption that different experimental conditions should exhibit equal amounts of kinase activity. Here we describe the development of analysis tools that reliably quantify phosphorylation of peptide arrays and that allow normalization of the signals obtained. We provide a method for intraslide gradient correction and spot quality control. We describe a novel interarray normalization procedure, named repetitive signal enhancement, RSE, which provides a mathematical approach to limit the false negative results occuring with the use of other normalization procedures. Using *in silico* and biological experiments we show that employing such protocols yields superior insight into cellular physiology as compared to classical analysis tools for kinome profiling.

Kinases, the regulators of cellular physiology, operate in strongly interconnected signaling networks^1^,^2^,^3^,^4^. While different techniques have been used to study kinase activity and protein phosphorylation^5^,^6^,^7^, the types of phosphorylation analyzed per experiment remain very limited and measurement of single kinases is insufficient to understand the complex regulatory processes at play. Parallel analysis of all kinases, the kinome, reveals more profound insight, and reduces the bias towards investigating known effects and interactions within the cellular signaling networks. Over the past years, peptide arrays have emerged as a powerful technique for such analysis^8^. Slide-based platforms include bovine peptide sequences^9^,^10^, 1196 peptides derived from the phosphobase repository^11^ of peptide kinase substrates^12^,^13^,^14^, and 1024 HPRD (Human Protein Reference Database)-based substrates^15^,^16^,^17^,^18^. However, quantification of the signals obtained and the subsequent normalisation of signals to correct for potential differences between the amount of input between experimental conditions remains challenging.

The analysis of radioactive peptide microarrays shows similarities to the well-established techniques used for quantification of DNA microarrays^19^, but a number of characteristics specific to peptide microarrays prompt for an adapted strategy for quantification and normalization. These include specific side-effects, such as fuzzy spot boundaries and presence of artifacts, the lower number of probes on a peptide array, and the fact that kinase-catalyzed phosphorylation reactions are less specific, with some peptides annotated to more than one upstream kinase (summarized in Fig. 1). Thus, a dedicated analysis pipeline is urgently needed for quantification, quality control and normalization to provide the best starting point for interpreting complex activity-based profiling for kinase signaling networks.

Normalization is necessary to remove systematic technical variation between array intensities, and allows comparison between different samples or days. Median-centering or quantile normalization, used in procedures for gene expression arrays^20^,^21^,^22^, are based on the assumption that different conditions yield identical intensity distributions^21^. This assumption does not always hold in peptide microarrays. The number of features on peptide microarrays is 10–100 times lower, greatly reducing the buffering capacity of the spots that are not affected by differences in experimental conditions. Also, depending on array content, a large fraction (5–20%) of the substrates might be differentially phosphorylated, with a bias towards increased phosphorylation in disease or upon stimulation. Furthermore, typically 50–80% of the substrates are not phosphorylated in either one or both experimental conditions. No housekeeping kinase with constant activity and high specificity is known, precluding the use of such a control for normalization. Indeed this is also not to be expected, as a regulator which is kept at constant activity has no purpose. The consequences of a change in intensity distribution for commonly used normalization procedures are illustrated using *in silico* generated data in Suppl. Fig. 1a–d.

Here we describe a two-step procedure in which we 1) present a novel method for intra-array normalization to correct for uneven signal distributions within a single array; and 2) present a novel interarray normalization method, based on selecting a subset of the data for normalization purposes. We explore the usefulness of this procedure with both an *in silico* experiment and a biological experiment with bone marrow specimens from patients with myelodysplastic syndrome (MDS).

## Results

### Prerequisites for array normalization: image processing and quality control

Active kinases (from a cell lysate) and radioactive ^33^P-γ-ATP, when applied to a peptide microarray, will phosphorylate specific peptides. Substrates that have been phosphorylated with a ^33^P-labeled phosphate group emit β-radiation onto a phosphorscreen, leading to a Gaussian excitation pattern (Suppl. Fig. 2a,b, array from a biological experiment). Spots in radioactive microarrays have inherent fuzzy boundaries between spot pixels and background pixels. An image enhancement step was introduced to simultaneously decrease noise, sharpen spot boundaries and increase contrast (Suppl. Fig. 2c,d). The enhanced images enable automated gridding and extraction of quality control (QC) parameters (see below) for individual spots. Please note that biological analysis of signal intensities uses data extracted from the raw image rather than these enhanced images.

Mild protein extraction methods necessary to preserve enzymatic activity unavoidably entails the formation of particulate structures that aspecifically bind radioactivity, provoking artifacts. Some of these artifacts are easily recognizable to the human eye, though can confound results if not removed automatically by image analysis software (for examples seen in a biological experiment see Suppl. Fig. 3). A successful approach for analysis of expression arrays is data quality-based flagging and subsequent exclusion of suspicious data^23^.

Using the enhanced image QC parameters can be calculated for each spot, based on distributions and patterns of pixel intensities. QC flags can be assigned to each spot, allowing the user to discard unreliable spots. When the intensity does not exceed the background, a spot is reliably not phosphorylated and will be called a off-spot. When a high variation in background pixel intensities is present, off-spots give a wider range of net intensities due to random fluctuations in pixel intensities. When the measured intensity is high, but no round object is detected at the expected positions, the signal is likely to be an artifact and the spot is flagged, i.e. it fails the QC. Supplementary Fig. S3 shows how such automated QC flagging results in selection of spots that are either reliably ‘on’ or reliably ‘off’.

Noise is inherently present in microarray data and diminishes the quality of analysis results^24^. When the total number of substrates per array is small, these off-spots can have a high impact on normalization procedures. In gene expression arrays, typically ~30% of spots show no signal, and are often excluded from further analysis. Peptide array experiments commonly display 50–70% off-spots (in our experience), and these are often not affected in the same way as phosphorylated spots by systematic inter-array variation. Hence we chose to employ normalization procedures that selectively use phosphorylated substrates to achieve more effective intra-array and interarray normalization.

### Intra-array normalization

The enzymatic nature of the peptide microarray assays in combination with the peculiarities of Michaelis-Menten biochemistry can cause small differences in kinase concentrations to produce significant intensity gradients over the arrays^24^. Concentration differences could be caused by uneven loading of the sample or by spatial gradients in the amount of peptide present on the array. These intensity gradients compromise data quality, which is not restored by standard normalization techniques. A standard 2D lowess correction (locally weighted scatterplot smoothing) is not feasible, because the correction is hampered by the low number of substrates and the large fraction of off-spots.

By using the three triplicate sets of substrates on the same array we developed a method for intra-array normalization that reduces the detrimental effect of intensity gradients (Fig. 2, examples of data sets with gradients are supplied in supplementary material S1). This gradient correction is performed using only spots that meet the QC criteria, excluding spot artifacts and off-spots. The remaining spots are used for a local median centering step, comparing the *N* (default: *N* = 20) nearest spots on the array (see methods).

### Interarray normalization

After intra-array correction of gradients, data are subjected to interarray normalization. We propose a novel normalization technique denoted repetitive signal enhancement (RSE), comprised of the following steps:

1. Local median-centering of the data using only spots that meet the QC-criteria in both conditions. For each spot location again the *N* (default: *N* = 20) nearest spots are used for this median-centering, now across the conditions from the different arrays.
2. Identification of spots potentially affected by the experimental condition. These spots are excluded during further normalization steps. We use quasi-stringent t-testing (p < 0.1) (using the replicates on the array) of QC-criteria fulfilling spots, excluding spots whose p-value indicates a statistically significant intensity difference.
3. Local median-centering of the data using only spots that meet the QC-criteria and that were not designated for exclusion in step 2.

Steps 2 and 3 are carried out iteratively until the set of spots stabilizes. Note that in every cycle, step 3 is based on the spots that meet the QC-criteria and the spots identified in the last iteration of step 2. This means that spots that are excluded early in the process might be used again later on and *vice versa*. Using RSE the set of spots used for normalization becomes progressively enriched for spots not affected by the treatment, leading to an unbiased normalization of a data set (Suppl. Fig. 1e). This step is then repeated in an iterative fashion with exclusion of spots that are significantly affected (t-test, p < 0.10) between the conditions. This set of unaffected spots used for normalization converges within 20 steps and typically consists of 70–90% of the complete set of spots.

### *In silico* validation of RSE

To compare the different normalization methods, we simulated a virtual biological experiment. Microarray results (each slide consisting of 3 sets of 1024 spots in a 32 × 32 layout (cf. Fig. 2)) were simulated for eight virtual patients. For each patient, a slide with a control condition and a slide with a treatment condition, e.g. a growth factor, was generated (experimental set-up and normalizations: Supplementary Fig. S4), with treatment activating two downstream pathways, consisting of 18 kinases with 197 downstream spots (parameters experiment: Supplementary Table S1). The experiment was performed with variations in i) effect size, ii) gradient strength, iii) percentage of induced spots, as a fraction of the total of 1024 spots, and iv) number of off-spots (see methods). As the induction of spots was artificially controlled, the outcome of each t-test could be unequivocally classified as: true positive, false positive, true negative or false negative. The results of this classification were used to construct Receiver Operating Characteristics curve, or ROC curves (Fig. 3). An ROC curve gives a graphical representation of the performance of the classification across all decision thresholds. The ROC curve can be summarized into a single statistic, the area under the ROC curve (AUC). Figure 3a shows ROC curves for the 8 virtual patients for different magnitudes of the treatment effect, demonstrating that more true positives are predicted with less false positives when treatment has a larger effect on spot intensities. In the absence of intraslide gradients and array effects (Fig. 3b), with only intraslide gradients (Fig. 3c), and with both intraslide gradients and array effects (Fig. 3d), AUCs decrease. These data show the detrimental effects of intraslide gradients and array effects on classification, signifying the need of normalization to correct for these effects.

Next, we compared the performance of different normalization procedures, i.e. interarray median centering over all spots without QC flagging, quantile normalization^20^ and RSE. With increasing effect size of experimental treatment, the AUC of all normalization methods increases. RSE corrects gradients and filters out the induced spots and elicits the best performance (Fig. 4a). Upon increasing the intraslide gradient strength, the necessity of gradient correction becomes more and more clear.

When uncorrected raw data are used as an input for subsequent interslide normalization, classification performance is substantially lower than after intra-array normalization in which the intraslide gradient correction was performed, but without RSE (Fig. 4b). When the percentage of induced spots increases, these spots have an increasing influence on the overall distribution of spot intensities, leading to a decreasing performance of median-centering and quantile normalization. Contrarily, RSE effectively filters out most of the effect of induced spots on normalization and shows a steady performance over a range of induced spots (Fig. 4c). Intraslide gradient correction and RSE normalization are both median-centerings that are performed locally. Large numbers of off-spots lead to an increase of the area that is used to find the *N* nearest spots used for normalization, which intuitively could affect the success of local normalization methods. The normalization methods we propose perform robustly over a large range of spots present on the array, i.e. even a large fraction of off-spots does not have a negative influence on classification performance (Fig. 4d).

Theoretically, as RSE relies on unaffected spots, an experimental setting where too many spots are induced by the experimental treatment might result in a loss of available spots for RSE and reduced efficacy of the normalization procedure. For kinome profiling arrays it is common for 5–20% of spots to be affected, and in our experience the number of spots affected rarely exceeds 40%, leaving enough unaffected spots to perform RSE normalizations. To test what would happen when over 50% of spots are induced, we extended the experiment performed in Fig. 4c and added this figure as a supplemental Fig. 5. We conclude that RSE outperforms interarray median centering over all spots without QC flagging and quantile normalization when less than 50% of spots are affected, but that gradient correction will have to be performed regardless of the normalization method.

### Using pathway analyses to correct for small deformations in data

We hypothesize that signaling pathway analyses are more sensitive to differential kinase activity between conditions than single spot analyses, because small inductions in phosphorylation over multiple downstream substrates can become statistically significant when analyzed in combination. This sensitivity is likely to extend to small deformations in the data caused by median-centering and quantile normalization. To test this hypothesis, we performed a pathway analysis with the setup and the range of experimental conditions as presented in Fig. 4a–d, but now include biological effect size variation and technical effect size variation to make the study more realistic (methods).

Upon increasing the effect size of the stimulation, intensity distributions between conditions become progressively different. This increases the number of unaffected pathways wrongly classified as being significantly repressed after median-centering or quantile normalization, leading to reduced AUC values, and RSE is less affected by this because of the iterative selection of a stable set of spots to be used for analysis (Fig. 5a). The statistical power of a pathway analysis increases when a larger fraction of the spots annotated to a pathway react to the treatment. RSE normalization shows a clear trend towards near-perfect classification upon an increase in the fraction of induced spots (Fig. 5b). For median-centering and quantile normalization this increase is largely offset by the normalization bias that is introduced.

### RSE allows outstanding characterization of cellular physiology of myelodysplastic syndrome (MDS)

To illustrate the superior effect of RSE over existing normalization procedures we performed a biological experiment in which kinome profiles were generated from patients affected with myelodysplastic syndrome (MDS). This hematopoietic disorder is characterized by an impaired differentiation of myeloid cells, erythrocytes and/or megakaryocytes^25^. Different types of MDS vary in severity and are characterized by the percentage of blood progenitor cells (CD34^+^ blasts) in the bone marrow^26^, with high-risk MDS patients often progressing to acute myeloid leukemia^27^.

Quality of the kinome profiling arrays was high, with a mean correlation of the within-array triplicates of 0.85 ± 0.05. Nevertheless, gradient correction significantly enhanced the overall correlation (0.87 ± 0.03, p = 0.0028, paired t-test), especially in experiments with a relatively large technical gradient, i.e., slides from patient 4), (Supplementary Table S2). The maximum gradient effect of the individual slides is shown in Supplementary Table S3, and ranges between 0.4 and 1.5. At these values, significant improvement can be achieved using gradient correction and RSE (Fig. 4b).

The percentage of active (phosphorylated) spots present in both unstimulated and SDF1 stimulated conditions ranged from 31 to 70% between the four patients. The number of spots induced by SDF1 outnumbered the spots suppressed by treatment, with a net percentage of induced spots between 5.6 and 11.2%. *In silico* experimentation indicated that results of different normalization procedures start diverging from a 2% induction of spots (Fig. 4c), suggesting that this experiment may indeed benefit from RSE normalization. This was further supported by the effect size of stimulation of samples −2log effect size ranged from 1.5 ± 0.7 to 2.3 ± 1.1, a range in which gradient correction followed by RSE showed significant improvement over other normalization procedures in our *in silico* experiment (Fig. 4a). Thus, the biological experiment described here conforms to several criteria that suggest a potential benefit from RSE normalization following intraslide gradient correction.

We performed a pathway analysis in which spots pertaining to one of several well-defined signaling pathways were compared by t-testing between control and SDF1-treated conditions. After exclusion of artifacts and intraslide gradient correction, the 2log-transformed values were normalized using either quantile normalization or RSE, followed by pathway analysis. Upon quantile normalization of the data we observed a significant general upregulation of kinase activity associated with the PI3K-PKB-mTOR pathway, the mitogenic pathway, mitosis/DNA damage and immune-associated responses and stemness signaling (Table 1). Activation of these pathways fit well with the molecular background of high-risk MDS^27^. SDF1 has previously been shown to be a potent activator of PI3K and MAPK signaling^28^, and indeed targets of these pathways are significantly more phosphorylated upon SDF1 treatment of MDS CD34 + cells.

Quantile normalization of data also resulted in the detection of small but significant decreases in phosphorylation of substrates that are a target for receptor tyrosine kinase signaling, AKT and S6K signaling, as well as Lyn, ATM, and WNT signaling. In addition, an overall decreased phosphorylation of substrates of kinases involved in G-protein signaling and cytoskeletal rearrangement was observed. These data are not supported by the current biomedical literature, and do not appear to be biologically relevant. For instance, one of the best-described functions of SDF1 is the induction of migratory responses in hematopoietic progenitor and other cells^29^,^30^. In addition, this chemotactic agent is a ligand for the G-protein coupled CXCR-4 receptor, engagement of which is known to stimulate the production of inositol3-phoshate (IP3) and diacylglycerol, resulting in PKA and PKC activation^31^,^32^. It is therefore highly unlikely that ligation of SDF1 to its receptor would induce negative regulation of these pathways, and suggests that quantile normalization, as predicted by *the in silico* experiment (Suppl Fig. 1d) results in the unjust detection of negative associations.

We next subjected intraslide gradient corrected data to RSE normalization prior to pathway analysis. RSE normalization greatly reduced the number of negative associations as compared to quantile normalization in these data sets. Using RSE, we no longer observed an inactivation of AKT and S6K kinase activity, PKA and PKC substrates were no longer less phosphorylated upon stimulation, and no negative modulation of cytoskeletal signaling was observed. In fact, we now detected significant phosphorylation of migratory signaling (i.e. Rac-PAK target phosphorylation) upon SDF1 stimulation of cells, and observed a significant increase in Notch-associated signaling. While Rac signaling upon SDF1 stimulation of CD34 + cells has been shown before^28^, Notch signaling upon SDF1 stimulation is a novel connection. Thus, our data show that RSE can uncover novel potential signaling pathways, which of course require further validation.

## Discussion

Normalization is essential to correct for differences in protein and enzyme input. The latter is especially challenging when different amounts of extracellular matrix are present in different experimental conditions, or when differences in sample handling can cause changes in the amount of denatured enzyme. In addition, approaches such as personalized medicine require samples to be analyzed on different days, resulting in different specific activity and purity of radioactive ATP batches and changes in exact handling, reaction time and small differences in reaction temperature^33^. Hence, establishing robust normalization protocols is considered as a challenge of utmost importance with respect to development of enzyme activity-assessing arrays^34^. Moreoever, the observation that in most peptide arrays over half of the peptide spots are blank/empty necessitates thorough normalization of the obtained signals. Woodard *et al*. have described intra-array normalization for cases where the signal distribution of the raw signal intensities of ‘empty’ or blank’ spots displayed a normal distribution with a mean value of 1^35^.

In the work of Li *et al*., background subtraction is used to remove the effect of technical artifacts^10^. This however inflates the variance. Li *et al*. propose to correct for this with a variance stabilizing transformation. In contrast, in our method, we forego background subtraction, by directly identifying and removing the variance inducing technical artifacts altogether, thereby not only removing signal background effects (e.g. local artifacts) as is done by background subtraction, but also signal gain effects (e.g. gradients and array effects). In RSE variance stabilization is unnecessary, as variances of spot fold-changes are reduced by the normalization, but not rescaled with respect to the log-transformed raw data. In this way, the variance accurately reflects the underlying signal significance of each spot, and small remaining technical artifacts (e.g. between arrays) are not inflated for lowly-expressed spots, thereby not unduly affecting aggregate tests across pathways.

Here, we show that in cases where biological differences in kinase activities lead to different intensity distributions, existing methods such as median centering or quantile normalization introduce a bias in the data, causing the artificial apparent down-regulation of phosphorylation of many substrates as a consequence of the induction of a limited set of other substrates. We therefore explored the usefulness of iterative selection of phosphorylated substrates that are not likely to show significant change between different experimental conditions. Using an *in silico* dataset, our method (termed RSE) was shown to perform robustly over a range of experimental conditions. The gain in performance over other methods increases with increasing difference in intensity distributions, for example when more spots are induced, or when the treatment leads to a bigger increase in intensity. Any microarray experiment with a difference between the total amount of induction and the total amount of repression might benefit from a normalization that leaves out the affected spots. The normalization then uses the unaffected spots for estimation and correction for systematic technical variation between arrays. Using only unaffected spots loosens the underlying assumption for normalization from “biological differences do not lead to different intensity distributions” to “a large fraction of substrates/features on the array is unaffected by the biological treatment”. The latter assumption will hardly ever be violated for a gene expression microarray experiment and thus provides a more solid basis for normalization.

The validity of this supposition was demonstrated by the biological experiment in this paper. Quantile normalization was unable to reproduce these literature-validated events, and unaffected pathways deceptively seemed to be repressed when other pathways were induced. In contrast, pathway analysis using RSE-normalized data showed good correlation with biomedical literature, and identified potential new pathways that may play a role in MDS pathology and may provide novel avenues of investigation. In this experiment, RSE normalization allowed for a more sensitive retrieval of biological information. We showed that the higher the induction of effect, the more negative statistical associations are observed after median-centering or quantile normalization, as the whole array is falsely over-normalized. However, when two experimental conditions are similar, and only modest effect sizes are observed, the risk of over-normalization may be less pronounced, and either RSE or other normalization procedures may be applicable.

In this manuscript we described a method for intra- and inter-array normalization of signals on peptide arrays. However, for the interpretation of the data one has to keep in mind that differences in phosphorylation status of the kinome can be greatly influenced by rapid reprogramming of protein phosphorylation states during sample preparation or due to ischemia in biopsy samples, especially in solid tumors^36^.

Taken together, our study shows that a novel approach to quantification of radioactive peptide microarrays in combination with normalization using data-based selection of unchanged phosphorylations results in markedly superior analysis as compared to current protocols. Widespread implementation of the protocol described here or protocols encompassing similar strategies would constitute an important step forward in realizing the full potential offered by peptide array-based kinome activity profiling and will contribute to inter-experiment data portability.

## Methods

### Pepchip analysis of biological samples

Pepchip peptide arrays (Pepscan, Lelystad, The Netherlands) consisted of 1024 different undecapeptides (11-mers), providing kinase substrate consensus sequences across the entire mammalian kinome. On each separate carrier, the array was spotted three times, to allow assessment of possible variability in substrate phosphorylation. The final physical dimensions of the array were 25 × 75 mm, each peptide spot having a diameter of approximately 250 μm, and peptide spots being 560 μm apart. The specificity of the assay was previously shown with ^33^P-α-ATP^37^.

This study was approved by the ethical review board of Lee Moffitt Cancer Institute, IRB 106308, with written consent from patients. “Informed” consent was obtained from all subjects. The methods were carried out in “accordance” with the approved guidelines. Mononuclear cells were separated by density centrifugation on Lymphoprep (FreseniusKabi, Oslo, Norway) and CD34 + cells were isolated from frozen bone marrow from 4 MDS patients, using positive selection by EasySep magnetic sorting according to the manufacturers’ instructions. Cells were allowed to recover in Hematopoietic Progenitor Growth Medium (HPGM, Lonza, Allendale, NJ) for 30 minutes, and either stimulated with 100 ng/ml SDF1 for 1 minute of left untreated. Cells were resuspended in mPER containing HALT protease and phosphatase inhibitors (Thermo Fisher, Rockford, Il). After clearing of the lysates by centrifugation, 20 μl activation mix was added (50% glycerol, 70 mM MgCl_2_, 70 mM MnCl_2_, 400 μg/ml BSA, 400 μg/ml PEG800, 2 μl ^33^P-ATP [PerkinElmer]) and samples were incubated at 37 °C on Pepchip arrays for 2 hours in a humidified incubator. Slides were washed in PBS + 1%Tween, in 2 M NaCl + 1%Tween and again in PBS + 1%Tween at 50 °C under continuous agitation. Slides were rinsed with dH2O and airdried, after which the phosphorimager screen was exposed to the arrays for 72h. Images were analyzed as described below.

### Image analysis

The image analysis procedure below was implemented in Matlab 7.11.0 (Mathworks, Natick, Massachusetts, U.S.A.). Matlab code is partly specific to the Pepchip array layout and is available upon request. For easy analysis of peptide arrays by researchers without the necessary background in computational analysis, we supplied executable programs that can be used on Mac, Windows and Linux operating systems (can be downloaded from: http://fmt.cs.utwente.nl/tools/ArrayAnalysis).

Before further analysis, pixel intensities were transformed according to equation 1 to reverse the effect of the square root transformation that was carried out by the phosphorimager (Molecular Dynamics)


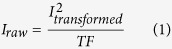



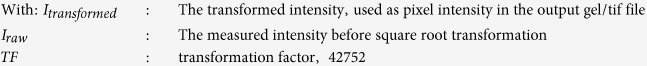


With: *Itransformed*: The transformed intensity, used as pixel intensity in the output gel/tif file

*Iraw* : The measured intensity before square root transformation

*TF* : transformation factor, 42752

A sequence of image processing operations was performed to reduce noise and increase contrast (Suppl. Fig. 2). After smoothening of the image with a median filter, a Laplacian of Gaussian filter (also known as Mexican Hat or Ricker wavelet) was applied to enhance round objects with the right size and a Gaussian signal distribution (Suppl. Fig. 2b,c). Spots are defined as round when the distance between two weighted centroids of the spots are within a radius of 250 μm, and when the shape ratio of the spot (length of longest axis divided by the length of the shortest axis) is smaller than 1.6. This operation sharpens spot boundaries. Morphological opening was used to remove small artifacts (<100 μm) and finally the image was smoothened again.

The image is thresholded at the 87^th^ percentile and is automatically rotated within a 3 degrees range to align the image vertically, a maximum of 3 degree rotation was sufficient in our experiments. This procedure assumes that the image is already roughly in a straight position, and relies on finding the orientation in which the spots are optimally aligned in vertical columns. The user is then asked to mouse-click on the boundaries of the array area that contains the spots. Based on this user-indicated spot area, locations of individual blocks of substrates are located. Each of these 8 × 8 blocks was spotted by a single pin of a multi-pin spotting tool.

Subsections of the array image that contain a single block of substrates are used to position a predefined ideal 8 × 8 grid on that block. This subsection is thresholded at the 96^th^ percentile for bright spots and at the 85^th^ percentile for weak spots. Round features of the expected size are detected and used for grid positioning. Artifacts are removed before gridding. The grid position and dimensions are optimized, such that it results in the highest overlap with the remaining objects. Bright spots are weighted 3 times stronger than weak spots. This optimization procedure is needed since both the spotting procedure and scanning of the phosphorscreen can introduce deviations in the size and position of these subgrids.

Image analysis definitions, used for the section below:

**Foreground signal:** Biologically relevant signal due to specific interaction between the sample and array features

**Background signal:** Noise, due to aspecific interaction between the sample and the array

**Net intensity:** Signal intensity after background subtraction

**Detection limit:** The lowest foreground intensity that can still be measured reliably

### Quantification of spot and background intensity

Individual spot positions were based on the grid position information and refined within a 100 μm (2 pixels) range. For each spot a circle shaped pixel selection (Ø = 250 μm, 21 pixels) is used to determine the mean spot intensity. Local background intensity is measured in a square range of 550 μm around the spot center. Pixels within a circular range of 275 μm from the spot center were excluded from the background to avoid confusion with spot pixels. Large artifacts and pixels originating from overly large spots were also excluded from the background measurement. Median background intensity was subtracted from the mean spot intensity to obtain the net spot intensity, which was subsequently log2 transformed.

### Quality-based flagging

Each spot was assigned a number of quality flags as a measure of spot reliability. To assign these flags, pixel areas surrounding each spot were analyzed for the distribution of pixel intensities. All flags are either 0 (reliable spot according to that flag) or 1 (unreliable spot according to that flag).

- Artifact: A large artifact (stripe, mark, blemish) shadows the spot and prevents reliable measurement
- Overshine: determines whether the presence of directly neighbouring bright spots is the cause of a spot intensity that is higher than the background. To be flagged for overshine, pixel intensity from a neighbouring spot must be uniformly decreasing into the spot area and the pixel intensity must be higher than the background intensity +3 * standard deviation.
- Kolmogorov Smirnov (KS): determines whether the distribution of spot pixels is sufficiently different from the distribution of local background pixels (p < 0.01).
- No Contrast: pixel intensities in the enhanced image were all <0, or the net spot intensity was <0.
- Saturated: more than 3 of the 21 spot pixels are saturated, i.e. have a reverse transformed pixel intensity >95.000
- Shape: The square area of 500 μm × 500 μm (12 × 12 pixels) around the spot center is thresholded at the 80^th^ percentile. When a reliable spot is present, pixels that are larger than the threshold belong to the spot. When the aspect ratio (largest diameter/smallest diameter) of the spot is >1.6 (for intense spots with a log2-value >8 and >2.0 for weak spots with log2 values ≤8), the spot is flagged for being insufficiently round to be a reliable spot.
- Position: After the same thresholding procedure, the intensity-weighted centroid of the object made up by the 20% brightest pixels is determined. When this centroid is located more than a user-defined distance (default 100 μm) away from the grid, the spot is flagged for being unreliable due to a faulty position
- Overall: If any of the above flags marks the spot as being unreliable, it is flagged as “overall unreliable”.

### Intra-array normalization

**Figure 1:**
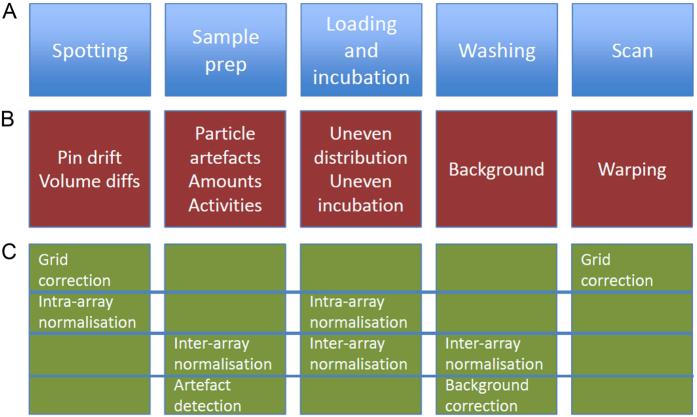
The problems hampering full exploitation of activity based profiling using peptide array analysis and the possible solutions to these problems pursued in present study. (**A**) Flowchart of peptide array-based kinome activity profiling process, starting with peptide spotting, sample preparation, incubation, washing, scan. (**B**) description of the issues created in each phase which need to be addressed in the analysis phase. (**C**) Solutions incorporated in the analysis pipeline to deal with these current issues in peptide array-based kinome profiling to allow meaningful comparison of different experiments.

**Figure 2:**
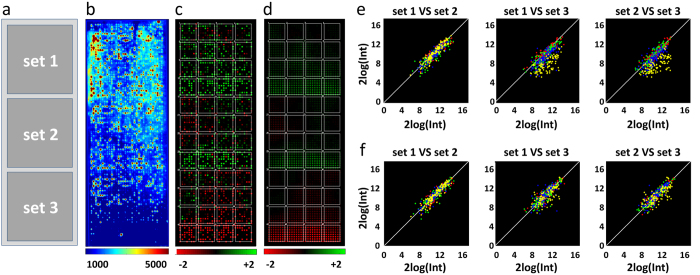
Presence of an intensity gradient on an example of a radioactive peptide microarray. (**a**) Slide layout, each set comprises 32 × 32 peptides. (**b**) False color image of an example array, showing low signal intensity in the bottom set. (**c**) Quantification of the relative spot intensities between the 3 sets. A green spot shows that a spot is more intense than the average intensity of the corresponding spots in the other two sets, a red spot is less intense. (**d**) For each spot position, the median of intensity deviations shown in (**b**) is taken over the 20 most closely located unflagged spots. Areas of increased (green) or decreased (red) intensity are clearly visible. (**e**) Scatterplots of the spot intensities between the 3 sets before gradient normalization. The intensities of the upper 8 rows in each set are plotted in red, rows 9–16 are plotted in green, rows 17–24 are plotted in blue and rows 25–32 are plotted in yellow. The right two panels compare set 1 and set 2 to set 3 respectively and show a markedly reduced intensity in the lower half of set 3, blue and (more noticeably) yellow dots. Correlations are 0.90, 0.67 and 0.68 for the three panels. (**f**) Scatterplots of the spot intensities between the 3 sets after normalization for the intensity gradients visible in (**c**,**d**). Correlation between the 3 sets is markedly improved by this normalization to 0.91, 0.81 and 0.87, respectively.

**Figure 3:**
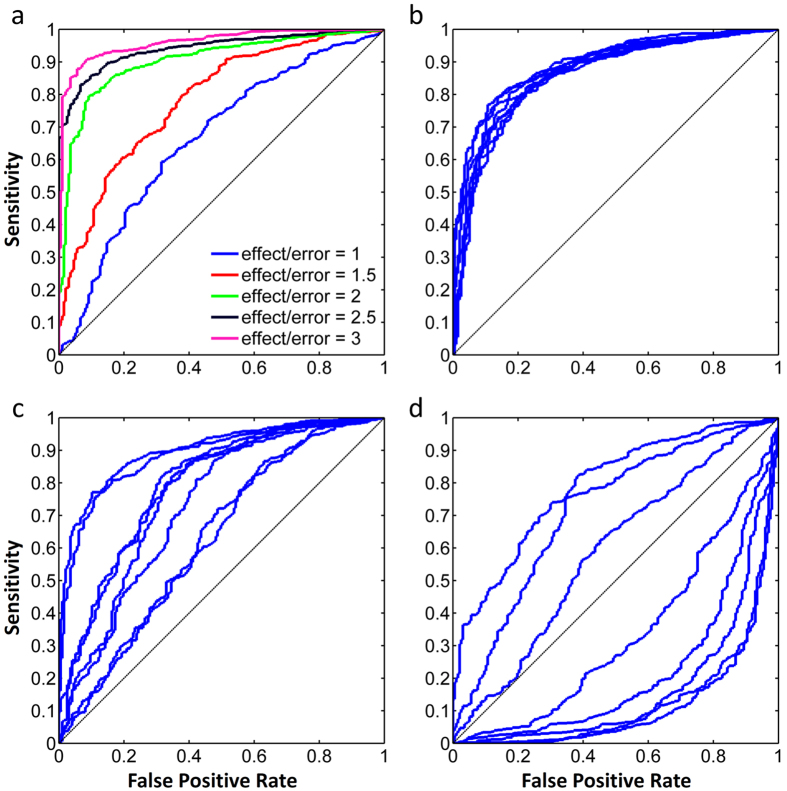
ROC curves for classification of single substrates in an *in silico* dataset as positives or negatives with a t-test (p < 0.05). 197 out of 1024 spots were induced for the following cases: (**a**) ROC curves for different ratios (induction effect)/(spot error) without intraslide gradients or array effects, each curve is a single patient. (**b**) ROC curves for 8 patients show the variation due to the random spot error, (induction effect)/(spot error) = 2, without intraslide gradients or array effects. (**c**) The presence of intraslide gradients (Suppl. Mat. 1) has a detrimental effect on spot classification as can be seen by the decreasing AUC. (**d**) When both intraslide gradients and array effects are present, the classification power decreases even further. AUC curves with AUC <0.5, i.e. curves under the line *y = x*, are seen when the net array effect between two slides approximately cancels the effect of substrate induction. In those cases a t-test preferentially classifies unaffected spots as (false) positives and affected spots as (false) negatives, leading to a classification that is worse than a random guess. (**c,d**) illustrate the need for both intraslide and interarray normalization.

The Pepchip peptide microarrays used in this study contain three replicate sets of 32 × 32 spotted substrates (Fig. 1a). Each spot on the array therefore has two replicate counterparts. Each log2 transformed net spot intensity is compared with these two replicates to find a deviation for each spot on the slide. These deviations are only calculated between reliable (i.e. unflagged) spots and are depicted in Fig. 1c. In the absence of systematic effects, this would yield a random distribution of negative and positive deviations, the magnitude of which would be largely determined by the random spot error. However, when areas are found where spots are systematically stronger or weaker than their replicates, a systematic effect is likely at play. To correct for this systematic effect, a local gradient is calculated for every individual spot, by taking the median deviation of the 20 most closely located unflagged spots, excluding the deviation of the spot itself. To this end, a circular region is defined that is centered around the spot. The radius of this circle is increased in a stepwise fashion until the minimum of 20 neighbouring spots is reached. The median deviation is calculated against both corresponding areas and then used to calculate a correction for each individual spot:


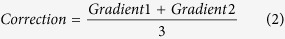


With:

- Correction: final correction value (log2 scale)

- Gradient1: median deviation against first set of replicates

- Gradient2: median deviation against second set of replicates

- 3: factor that ensures appropriate correction when each spot is corrected in by this method.

### Interarray normalization

Intra-array normalized intensity values are used as input for inter-array normalization. Slides were inter-array normalized per pair (with or without SDF1 from each patient) using RSE normalization. Spots that are flagged when either of the conditions are excluded from the normalization procedure. RSE takes an approach similar to intra-array normalization. Instead of three sets that are compared to each other, the interarray normalization compares the average of the triplicate spot intensities on one slide to the average of the triplicate spot intensities on the other slides, as described previously.

### *In Silico* validation of RSE

All intensity values are expressed as a log2 transformed dimensionless value. Each spot intensity was the sum of a i) random basic intensity, normally distributed (*μ* = 10, *σ* = 1.5); ii) random value for inter-patient biological variation, normally distributed (*μ* = 0, *σ* = 0.5); iii) treatment effect for induced spots; iv) local intraslide gradient effect, one of 15 possible 96 × 32 gradient arrays was randomly added to each slide; v) uniformly distributed random array effect from the range [−2, 0]; and vi) random spot error, normally distributed (*μ* = 0, *σ* = 0.4).

For each patient, a t-test was performed for each substrate to examine statistical significance between the triplicate values of the two conditions.

### Pathway analysis

**Figure 4:**
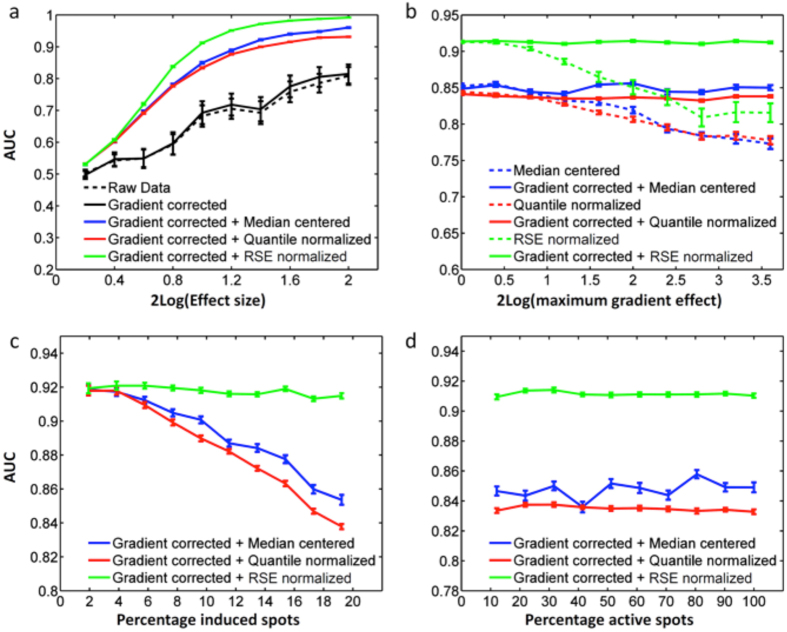
Performance of different normalization techniques across a range of experimental conditions, measured by the AUC of ROC curves. (**a**) AUC increases with increasing effect size of experimental treatment. (**b**) Upon increasing intraslide gradient strength, intraslide gradient correction as a first step gives an increasing contribution to classification performance. (**c**) Median-centering and quantile normalization show a decreasing performance upon increasing the fraction of induced spots. This is the percentage of all 1024 substrates. (**d**) Median-centering, quantile normalization and RSE normalization all show a stable performance over a range of active spots. All data points in (**a**–**d**) are the average of 8 patients control vs treatment). Error bars represent the SEM. Parameter settings for each of these *in silico* experiments are given in Suppl. Table 1.

**Figure 5:**
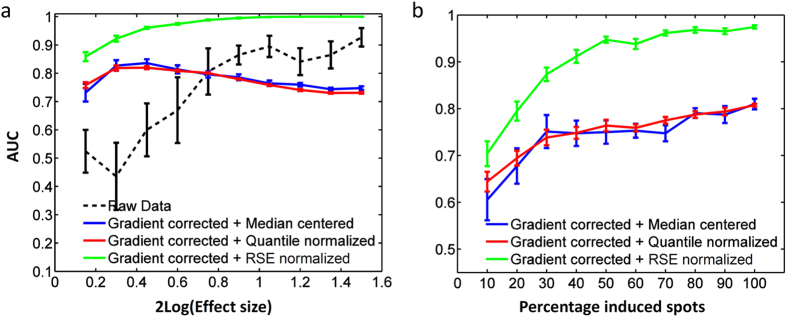
Performance of different normalization techniques across a range of experimental conditions, measured by the AUC of ROC curves of a pathway analysis. (**a**) AUC increases with increasing effect size of experimental treatment for raw data or RSE normalization, but drops after an initial increase for median-centering and quantile normalization. (**b**) When a larger fraction of spots that is assigned to a pathway is induced, the AUC increases, with RSE increasingly outperforming median-centering and quantile normalization. This is the percentage of spots that is annotated to an induced pathway, i.e. 100% means that 197 spots out of 1024 spots are induced. AUC values are the average performance of 10 experiments with eight virtual patients each (control vs treatment). Error bars represent the SEM. Parameter settings for each of these *in silico* experiments are given in Suppl. Table 1.

Pathway analysis was performed with the same setup and same range of experimental conditions presented in Fig. 4a–d. To make the study more realistic two additional sources of variation between spots were introduced:

1. Biological effect size variation, normally distributed (μ = 0, σ = 0.4), accounting for the fact that different patients can react differently to a growth factor.
2. Technical effect size variation, uniformly distributed between [−0.2, 0.2] (between [−0.33*E, 0.33*E] when the effect size was varied, Fig. 5a). This accounts for the fact that spotted peptides can react with different kinetics to activation of the same kinase.

RSE-normalized values were subsequently used for pathway analysis, in which spots pertaining to one of several well-defined signaling pathways were compared by paired t-testing for the individual conditions. Annotation of the spots to upstream kinases was taken as described by Pepscan (Lelystad, The Netherlands), based on the human protein reference database (www.hprd.org), and manually validated by literature searches. All spots annotated to one kinase were combined to present signaling networks, allowing for a more robust analysis of pathway activation as compared to individual spot analysis. Spots for which the annotation was not clear were not included (‘other’ in Table 1).

## Additional Information

**How to cite this article**: Scholma, J. *et al*. Improved intra-array and interarray normalization of peptide microarray phosphorylation for phosphorylome and kinome profiling by rational selection of relevant spots. *Sci. Rep*. **6**, 26695; doi: 10.1038/srep26695 (2016).

## Supplementary Material

Supplementary Information

Supplementary Table S1

## Figures and Tables

**Table 1:**
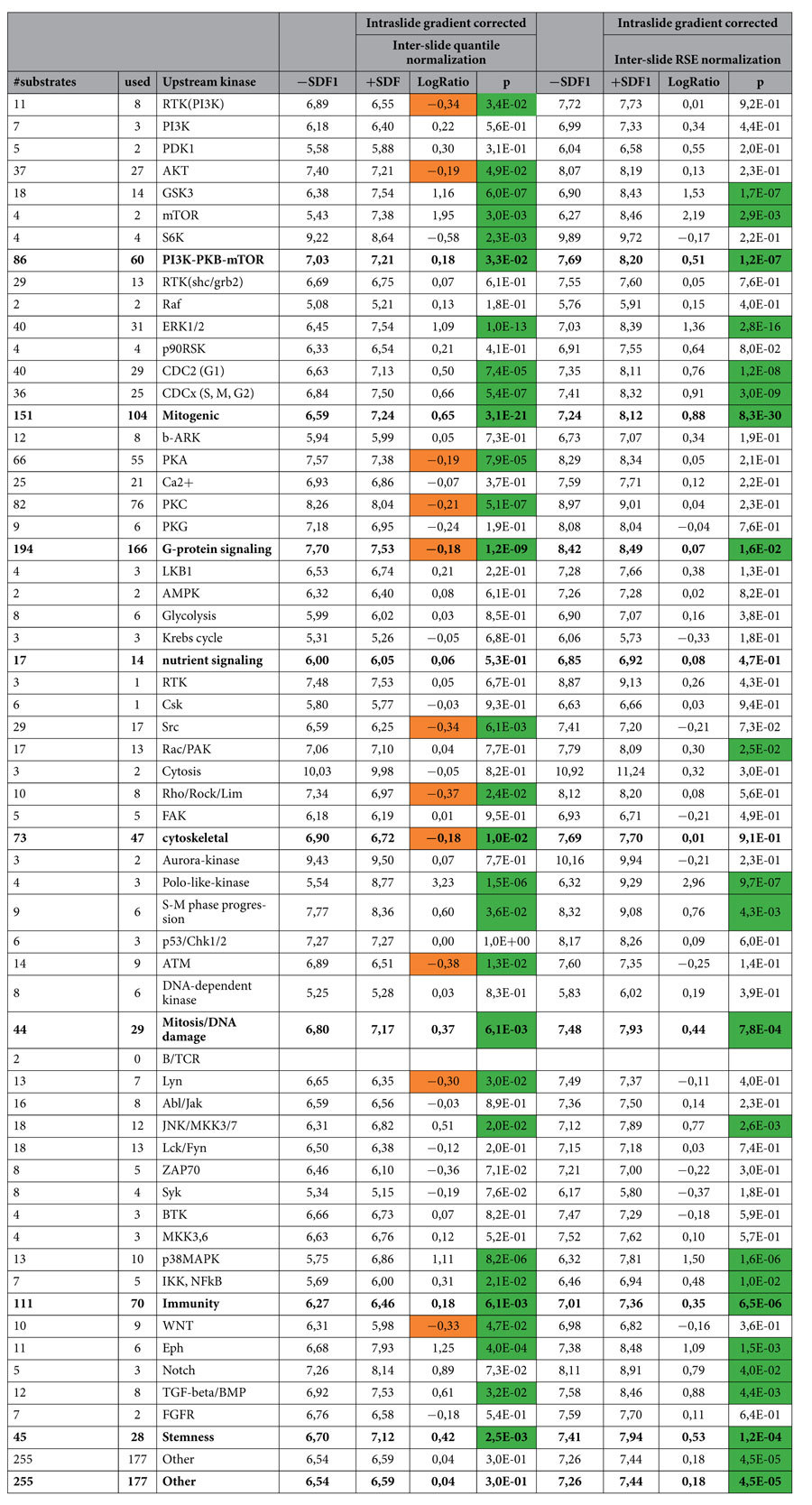
Pathway analysis of SDF1 induced changes in kinome profiles in 4 high risk MDS patients, analysed using either quantile or RSE normalization.

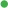
 Significant changes upon SDF1 treatment (paired T-testing); 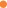
apparent repression of activity in quantile normalised setting.
