# Supplementary material for: Improved intra-array and interarray normalization of peptide microarray phosphorylation for phosphorylome and kinome profiling by rational selection of relevant spots
Source: Sci Rep. 2016 May 26;6:26695. doi: 10.1038/srep26695 (PMC4881024; doi:10.1038/srep26695)
Supplement: Supplementary Information [file srep26695-s1.pdf]

Supplemental figures and tables for "Improved intra-array and interarray normalization of peptide microarray phosphorylation for phosphorylome and kinome activity profiling by rational selection of relevant spots"

Jetse Scholma[1], Gwenny M. Fuhler[2], Jos Joore[3], Marc Hulsman[4,5], Stefano Schivo[6], Alan F. List[7], Marcel J.T. Reinders[5], Maikel P. Peppelenbosch[2,*], Janine N. Post[1]

Contains:

Supplemental Figures 1, 2, 3, 4, and 5

Supplemental tables 1, 2, and 3

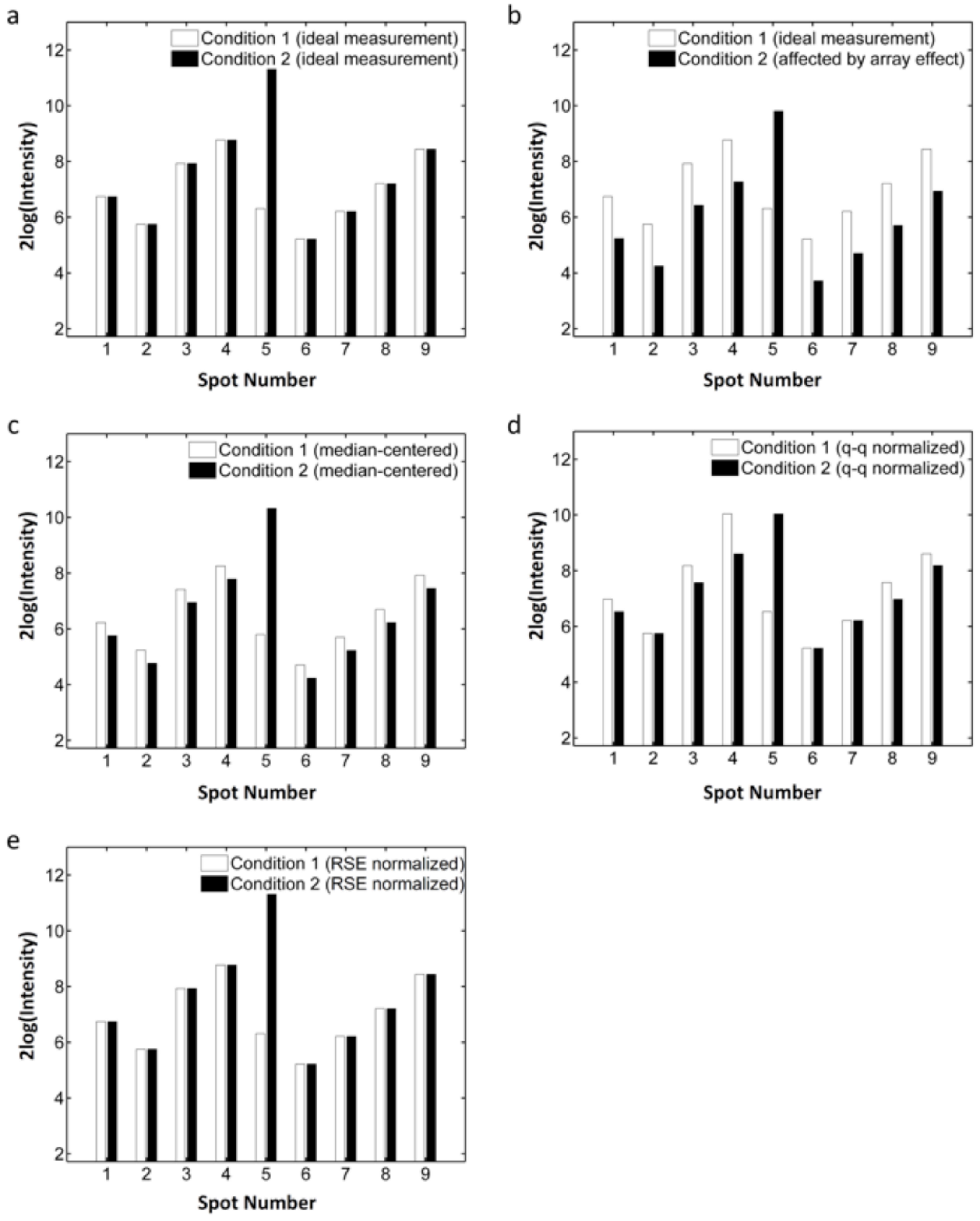

Supplementary Figure 1: Standard normalization techniques lead to data deformation in case of a large difference between experimental conditions. White bars indicate condition 1, which is without treatment, Black bars indicate a treated condition. This virtual treatment is the same for all normalization techniques. A) Randomly generated result for a hypothetical microarray experiment, with 9 spots per array. For simplicity,

measurement error is assumed to be zero. Spot 5 is strongly induced by this treatment condition (black bars), whereas the other spots are unaffected. B) The same result, condition 2 is affected by a technical variation, causing spot intensities to be systematically lowered. C) The same data as in b, after normalization using median-centering. D) The same data as in b, after quantile normalization. Both c and d show the introduction of a bias in the normalized results, where many of the unaffected spots have a lower intensity after normalization. This example serves to illustrate similar normalization effects that we observed on more realistically sized arrays containing several hundreds to thousands of spots. E) RSE normalized data, showing no bias in the analysis of the treatment effect.

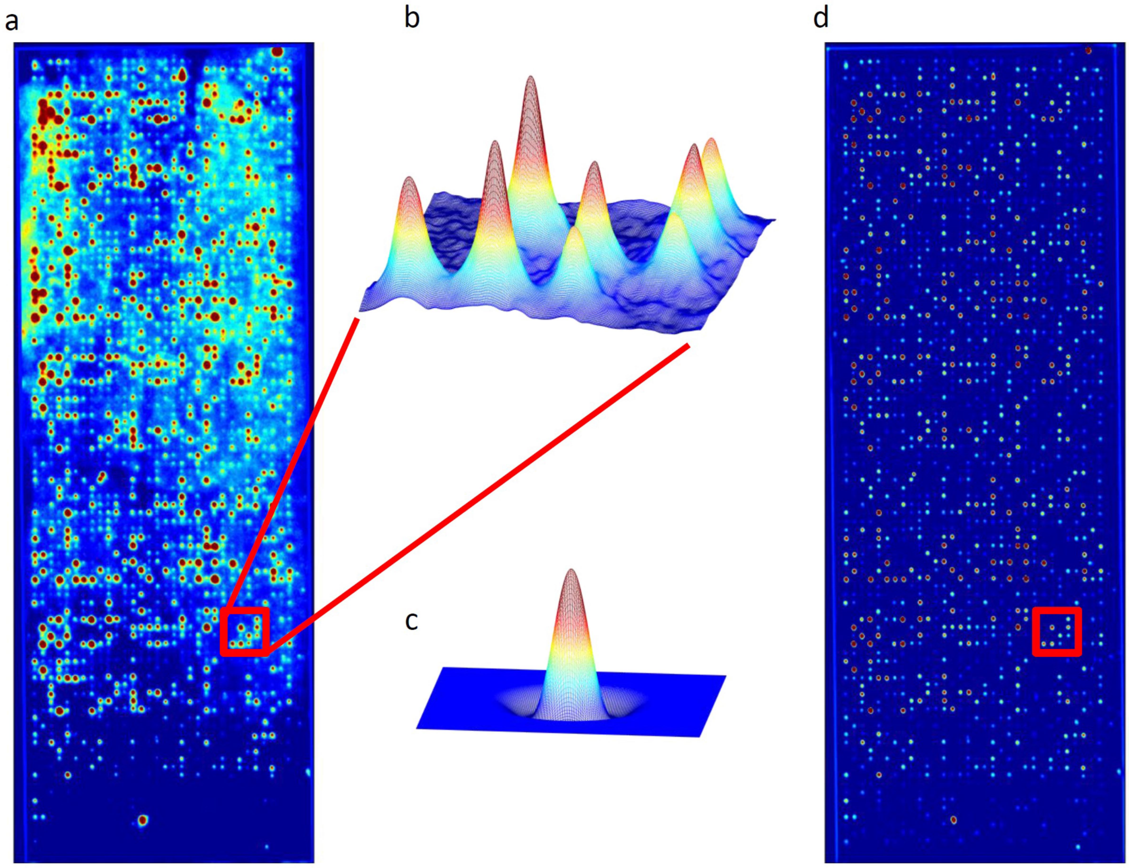

Supplementary Figure 2: Image enhancement leads to better defined spots and increased contrast. A) False color image of a radioactive peptide microarray (raw image). B) Mesh plot of a subsection of the array displayed in a), showing the gaussian distribution of pixel intensities for multiple spots. C) Mesh plot of the laplacian-of-gaussian filter ("Mexican Hat") that is used to enhance the raw image and to increase contrast. D) The corresponding array after image enhancement.

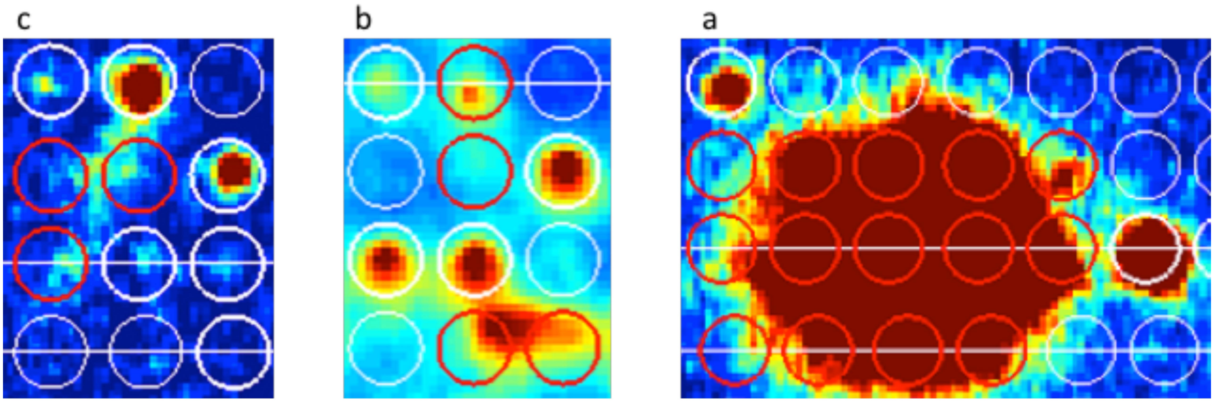

Supplementary Figure 3: Different types of artifacts are automatically detected to improve data quality by selectively discarding unreliable spots. A) A weak stripe is visible. Flags are indicated by red circles. B) The two flagged spots (red circles) at the top have the highest intensity located too far from the expected spot center (middle of the circle), the bottom two flagged spots show a not-round object that is not centered at a spot position. C) A larger blemish prevents reliable measurement of a number of spots.

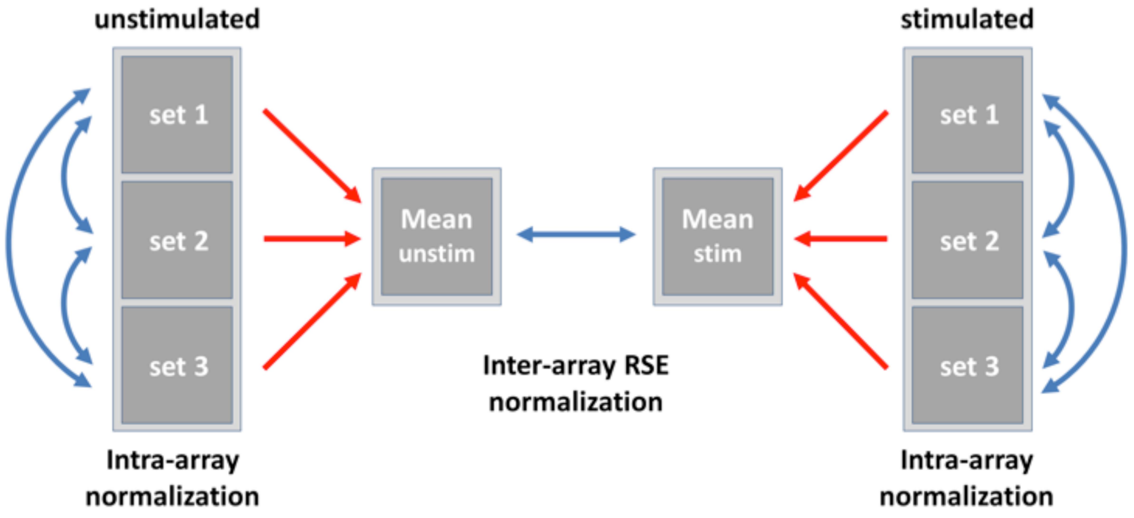

Supplementary Figure 4. Schematic representation of kinome analysis in which two experimental conditions are compared. This situation is applicable to both the *in silico* and the biological experiment presented in this study. Unstimulated and stimulated samples (i.e. SDF1 in the biological experiment) are applied to a kinome array, First, intraslide gradient correction is performed, thus improving the correlation between the triplicate substrates on a slide. Inter-slide normalization is performed on the mean of the substrate triplicates.

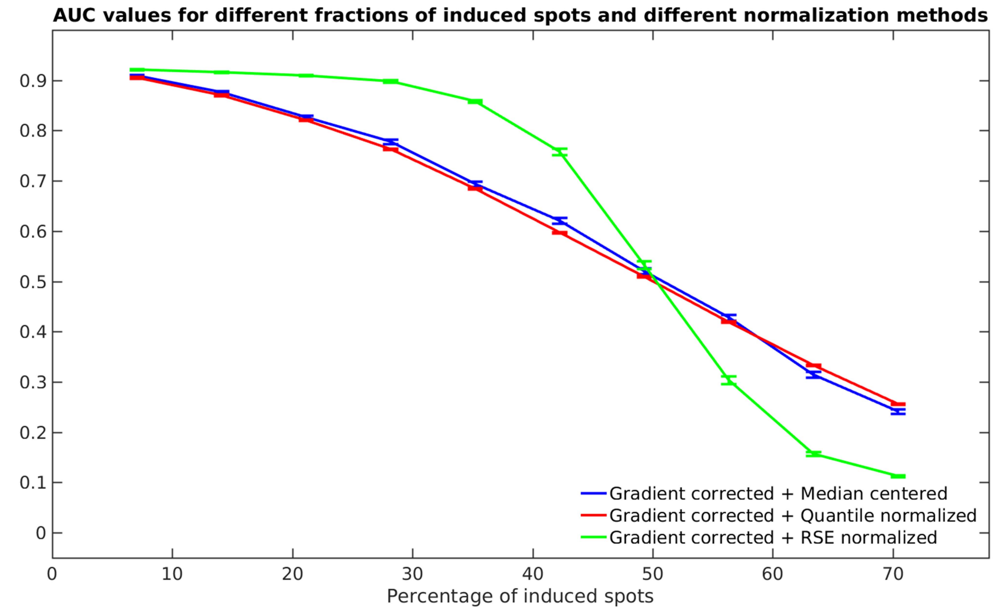

Supplementary figure 5. AUC values for different percentages of induced spots using different normalization methods. This is the percentage of all 1024 substrates. At a low number of induced spots, 5-40% RSE normalization shows a clear advantage over both Median-centering and quantile normalization, which show an initial decrease in performance upon increasing the fraction of induced spots. At >55% induced spots there seems no advantage of RSE over Median centered and quantile normalization. Gradient correction should always be performed. In our experience 5-20% of spots are induced over an array of 1024 substrates. At these low numbers performing RSE has a clear advantage.

Supplementary Table S1 parameters for *in silico* PepChip experiments

**The following parameters were used to create an *in silico* Pepchip experiment:**
**Slide layout:**
Each slide contains 1024 peptide substrates (32x32) in three triplicate sets (Fig 2a)
**Experimental design:**
Results were simulated for 8 patients, with 1 control slide and 1 treatment slide for each patient (16 slides in total)
2 pathways were assumed to be activated by the treatment, with a total of 18 kinases and 197 downstream substrates
Slides were randomly affected by an intraslide gradient, which was added to the spot intensities (Suppl. Mat. 1). The gradient ranged linearly from the maximum effect (-2 on a 2log scale) to 0.
Slides were affected by a uniformly distributed array effect, range [-2, 0], 2log scale
A normally distributed random error ($\mu = 0$, $\sigma = 0.4$, 2log scale) was added to each substrate intensity
Upon treatment, intensities of downstream activated substrates were increased with the treatment effect (0.8, 2log scale)
**Variations on this design for figure 4:**
Fig 4a: Treatment effect size was varied from 0.2-2 in steps of 0.2 (on a 2log scale)
Fig 4b: Maximum gradient effect was varied from 0 - 3.6 in steps of 0.4 (on a 2log scale)
Fig 4c: A varying fraction (10% to 100%) of the downstream 197 substrates was induced, resulting in roughly 2-20% of 1024 spots on the array being induced
Fig 4d: A varying fraction of spots was selected to be completely not responsive, i.e. off-spots. Of the 1024 spots, 0-900 spots were set as off-spots (both induced substrates and not induced substrates were selected as off-spots), leaving 10-100% of all 1024 spots active.
**Further variations on this design for figure 5:**
For the pathway analysis, three further variations were introduced:
1: Biological variation between patients, normally distributed ($\mu = 0$, $\sigma = 0.5$, 2log scale)
2: Variation of the effect of the treatment on induced spots, uniformly distributed [-0.33*Effect_Size, 0.33*Effect_Size], 2log scale) (not all downstream spots react equally strong on the treatment)
3: Biological variation of the effect of the treatment on induced spots, normally distributed ($\mu = 0$, $\sigma = 0.3$,

2log scale) (Patients can react differently to the same treatment)
For figure 5a: mean Effect_Size was varied between 0.15 and 1.5 in steps of 0.15 (2log scale)
For figure 5b, mean Effect_Size was set at 0.6 and a varying percentage (10-100%) of 197 downstream spots was induced

Supplementary Table S2. Characteristics of arrays of biological experiment

	Patient			
	**1**	**2**	**3**	**4**
**2log Effect size (mean +/- stdev)**	2.0 +/- 0.9	2.0 +/- 1.0	2.3 +/- 1.1	1.5 +/- 0.7
**stdev of 2log (Spot Error) - SDF1**	0,6	0,6	0,5	0,4
**stdev of 2log (Spot Error) + SDF1**	0,5	0,5	0,5	0,4
**# active spots (overlap between -SDF1 en + SDF1, unflagged spots on both slides)**	323	505	559	713
**% of active spots (# active spots/1024*100)**	31,5	49,3	54,6	69,6
**# geinduced spots (p < 0.05)**	42	69	77	135
**# repressed spots (p < 0.05)**	24	22	15	55
**Netto % induced spots (= (Ind - Repr)/Tot*100%)**	5,6	9,3	11,1	11,2
**Max Gradient effect - SDF1**	0,4	0,9	0,7	1,5
**Max Gradient effect + SDF1**	0,8	0,9	0,9	0,9

Supplementary Table 3. Intraslide spot correlations before gradient correction, after gradient correction, and the difference (the green-scale indicates the beneficial effect of gradient correction).

Correlations before gradient correction

**Patient**	**1**	**1**	**2**	**2**	**3**	**3**	**4**	**4**
**Treatment**	**-SDF1**	**+SDF1**	**-SDF1**	**+SDF1**	**-SDF1**	**+SDF1**	**-SDF1**	**+SDF1**
**Corr1_2**	0,81	0,88	0,84	0,91	0,90	0,89	0,89	0,88
**Corr1_3**	0,79	0,81	0,79	0,84	0,88	0,86	0,71	0,83
**Corr2_3**	0,84	0,87	0,83	0,87	0,90	0,87	0,74	0,85

Correlations after gradient correction

**Patient**	**1**	**1**	**2**	**2**	**3**	**3**	**4**	**4**
**Treatment**	**-SDF1**	**+SDF1**	**-SDF1**	**+SDF1**	**-SDF1**	**+SDF1**	**-SDF1**	**+SDF1**
**Corr1_2**	0,82	0,88	0,85	0,91	0,91	0,90	0,90	0,89
**Corr1_3**	0,81	0,83	0,79	0,87	0,89	0,87	0,85	0,88
**Corr2_3**	0,84	0,87	0,83	0,89	0,91	0,89	0,87	0,88

Difference (after - before)

**Patient**	**1**	**1**	**2**	**2**	**3**	**3**	**4**	**4**
**Treatment**	**-SDF1**	**+SDF1**	**-SDF1**	**+SDF1**	**-SDF1**	**+SDF1**	**-SDF1**	**+SDF1**
**Corr1_2**	0,00	0,00	0,01	0,00	0,01	0,00	0,01	0,01
**Corr1_3**	0,01	0,02	0,00	0,03	0,01	0,02	0,14	0,05
**Corr2_3**	0,00	0,00	0,01	0,01	0,01	0,02	0,12	0,04
